# Improving analgesia provision for sheep: An analysis of farm medicine records and attitudes towards pain relief on sheep farms in Northern Ireland

**DOI:** 10.1002/vro2.75

**Published:** 2023-10-23

**Authors:** Paul E. Crawford, Kim Hamer, Fiona Lovatt, Malgorzata C. Behnke, Philip A. Robinson

**Affiliations:** ^1^ Department of Animal Health, Behaviour and Welfare Harper Adams University Shropshire UK; ^2^ School of Biodiversity, One Health and Veterinary Medicine, College of Medical, Veterinary and Life Sciences University of Glasgow, Garscube Campus Glasgow UK; ^3^ School of Veterinary Science Sutton Bonington Campus University of Nottingham Nottingham UK; ^4^ Flock Health Ltd., Egglesburn Farm, Eggleston, Barnard Castle Durham UK; ^5^ Harper & Keele Veterinary School Harper Adams University Campus Shropshire UK; ^6^ Keele University Staffordshire UK

## Abstract

**Background:**

Management of pain is critical to improve the welfare of farmed livestock and meet consumer expectations. There is limited published information about the use of analgesic drugs in the sheep sector.

**Methods:**

A mixed‐method approach was followed. The range of analgesic drugs used on 52 Northern Irish sheep farms was determined through analysis of medicine purchase records. Through interview and discussion groups, with both farmer and veterinarian participants, attitudes towards the use and adoption of such medicines were explored.

**Results:**

The use of non‐steroidal anti‐inflammatory drugs (NSAIDs) was widespread and highly variable. One‐third of farmers in the sample did not purchase any NSAID. Meloxicam was the most commonly purchased NSAID by mass (72%) and standardised dose (73%). During interviews and discussions, farmers outlined the benefits they saw in using NSAIDs and how veterinarians influenced their uptake of these medicines. Use of corticosteroid was evidenced on 50% of the farms that supplied medicine records for analysis.

**Conclusions:**

Veterinarians can influence farmers to adopt NSAIDs for the provision of analgesia in their sheep and farmers observed the benefits they delivered. However, many farmers are still to be reached with this message, perhaps due to being largely self‐sufficient and rarely engaging with veterinarians. Veterinarians have the opportunity to challenge farmers about the provision of analgesia, especially when farmers seek antibiotics for painful conditions such as lameness. Currently, the lack of an authorised product in the UK, with associated treatment guidance and industry promotion, may limit veterinarians’ confidence in prescribing drugs for pain control in sheep.

## INTRODUCTION

Pain in sheep is a complex sensory and emotional experience arising directly, following damage to tissue and secondarily to inflammation.^1^ Pain is influenced by previous painful experiences and social position within the flock.^1^, ^2^ In sheep farming, pain can arise from a wide range of disease and management‐related factors.^3^, ^4^

The importance of adequate analgesia has been recognised and is enshrined in legislation in many countries.^4^ The management of pain in livestock continues to come under increasing scrutiny from the public^2^, ^4^, ^5^; additionally, painful conditions, such as lameness, carry an economic cost to farmers.^6^ However, before pain can be effectively managed, those caring for sheep need to recognise its presence and appreciate the need for pain to be treated promptly and appropriately.^3^, ^4^ Although no universal, validated pain scoring system yet exists for sheep, previous reviews have highlighted subjective and objective tools that can aid in pain assessment^1^, ^2^, ^4^; while many of these are more suited to research settings,^7^ research continues to develop systems for on‐farm use.^8^ Industry bodies have prepared guidance notes for farmers^9^, ^10^ to make information on pain assessment accessible to them. Previous studies have highlighted that while both farmers and veterinarians are cognisant of and can identify procedures that may cause pain and can identify signs of pain, there is a variation in how participants in these studies rank the severity of the pain.^1^, ^11^ Furthermore, sheep are known to moderate their behavioural expression, which may mask signs of pain following disturbance or handling, influenced by the management system and prior experiences.^1^, ^12^, ^13^

All of the major classes of analgesic drugs have been shown to provide some level of analgesia in sheep.^2^, ^3^ Currently, however, in the UK, there are no products specifically authorised for use in sheep to provide long‐term analgesia.^14^ Under the ‘Cascade’ system,^15^ products authorised for use in other food‐producing animals may be prescribed for sheep, by a veterinarian, on a case‐by‐case basis for the protection of animal welfare. The lack of any non‐steroidal anti‐inflammatory drugs (NSAIDs) authorised for use in sheep in the UK has been identified as having major animal welfare implications. Calls have been made for novel approaches to the generation of the data necessary to prove the efficacy and safety of such products for use within the sheep sector.^16^ For cattle, NSAIDs have proven practical to administer and efficacious in providing long‐term analgesia when administered on‐farm by the farmer.^5^ However, there is evidence of underuse of these medicines in cattle and calls for further education to reinforce the benefits of timely analgesia.^5^

Corticosteroids (CSs), such as dexamethasone, but not NSAIDs, have been used for their anti‐inflammatory properties in a range of livestock species, including for provision of analgesia, despite potential side effects and limited evidence of clinical pain‐relieving properties.^17^, ^18^ Historically, evidence for any long‐term therapeutic benefit of CS has been limited to specific circumstances, at least in cattle.^19^

The metric mg/population‐corrected unit (PCU) was initially developed for comparing antibiotic use where flocks, breeds and production systems differed between nations.^20^ The PCU metric standardises antibiotic use based on standardised, averaged body masses for the class(es) of livestock under consideration. Subsequently, they have been utilised widely to quantify, compare and benchmark antibiotic use nationally, also as an indicator of antibiotic use on and between individual farms. To the best of the authors’ knowledge, no such work has been undertaken regarding analgesia provision in sheep.

This report presents a preliminary study that aimed to develop an understanding of the attitudes and behaviours of Northern Irish sheep farmers and their veterinarians towards NSAID and CS use in sheep. Additionally, it sought to identify stimuli to achieve wider use of pain relief in sheep and any barriers to increased analgesia provision in the sector.^4^, ^21^

## METHODS

A mixed‐method research approach was utilised. Data comprised records of medicines purchased over a 12‐month period, semi‐structured interviews with farmers and veterinarians, and discussion groups with participants drawn from the same sectors. Convenience sampling was used for selection of participants throughout each phase of data collection, with no restriction on how many elements a participant could contribute to. These data were collected as part of a wider programme considering medicine use and stewardship in the Northern Ireland sheep flock.

Medicine records were obtained from on‐farm records (eight farmers) or directly from the farmer's veterinary practice's sales records (50 farmers), with six farmers supplying records from both sources. All farmers were asked about their livestock enterprises. There were 13 (25%) sheep‐only livestock enterprises. During discussion with the remaining 39 (75%), their medicine purchases were allocated to each of their enterprises. Only data pertaining to the sheep flock were analysed. Medicine purchase data were transcribed into Microsoft Excel^22^ for analysis. Each flock's mean antibiotic consumption was calculated in mg/PCU using the University of Nottingham Antibiotic calculator.^23^ To contextualise the quantity of NSAID and CS purchased, flock size, total antibiotic purchased and antibiotic consumption, in mg/PCU, were used as denominators. Spearman's R was used to determine the significance of the relationship between pairs of variables.

Standardised, semi‐structured interviews and discussion groups were undertaken with farmers and veterinarians (Supporting Information S1). Electronic recordings or contemporaneous notes were taken from interviews and discussion groups. Following transcription, NVivo^24^ software was utilised during coding. The first author undertook all the medicine record analysis, interviewing and facilitation of discussion groups, and transcription. During interviews and discussion groups, participants were assured that their knowledge of sheep medicine was not being tested. They were not asked for the rationale behind specific treatments or dose rates. Where these were offered, the information was recorded. One follow‐up question addressing CS was sent to veterinarians by email (Supporting Information S1). After initial coding, sections relevant to pain management and the use of NSAIDs were collated and exemplar quotes were identified.

## RESULTS

### Medicine records

Three NSAIDs, meloxicam, flunixin and ketoprofen, and one CS, dexamethasone, were identified in the records. Meloxicam (72%) was, by mass of active component, the most purchased, followed by flunixin (14%), ketoprofen (12%) and dexamethasone (2%).

**FIGURE 1:**
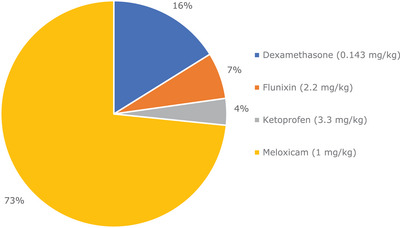
Relative proportions in standardised dose (based on the authors’ experience of clinical practice in the UK) for the single corticosteroid and three non‐steroidal anti‐inflammatory products identified in the 12‐month sample of medicine records supplied relating to 52 Northern Irish sheep farms.

To account for differing potencies and dose rates, the number of standardised doses purchased was calculated utilising dose rates the authors considered represented best‐practice clinical use in the UK: meloxicam 1 mg/kg, ketoprofen 3.3 mg/kg, flunixin 2.2 mg/kg and dexamethasone 0.14 mg/kg. This showed meloxicam was the most utilised product (73%), followed by dexamethasone (16%), flunixin (7%) and ketoprofen (4%) (Figure 1).

Medicine records revealed that 14 (33%) farmers had purchased no NSAID during the 12 months covered by the records supplied (Table 1). Of the 38 farmers who did purchase NSAIDs, 17 (30%) purchased 50 mL or more. The CS dexamethasone was purchased by 26 (50%) farmers, with half of these purchasing 50 mL or more. Both NSAID and CS were purchased by 23 (44%) farmers. Eleven (21%) farms purchased neither NSAID nor CS (Table S1).

**TABLE 1:** Frequency distribution of quantities by volume of each non‐steroidal anti‐inflammatory drug (NSAID) and corticosteroid (CS) product purchased by farmers, as identified in the 12‐month sample of medicine records supplied relating to 52 Northern Irish sheep farms.

	None	Less than 10 mL	10–50 mL	50–100 mL	100 mL and greater
NSAID					
Meloxicam	17 (33%)	16 (31%)	6 (12%)	2 (4%)	11 (21%)
Ketoprofen	50 (96%)	0 (0%)	1 (2%)	0 (0%)	1 (2%)
Flunixin	46 (88%)	3 (6%)	0 (0%)	1 (2%)	2 (4%)
CS					
Dexamethasone	26 (50%)	8 (15%)	5 (10%)	10 (19%)	3 (6%)

*Note*: In 11 of the records, no NSAID or CS purchases were identified; 24 records indicated purchase of both NSAID and CS. The majority of farms (94%) that purchased NSAID, purchased only one NSAID. Two (4%) farms purchased two different NSAIDs and one (2%) purchased all three of the NSAIDs identified.

**FIGURE 2:**
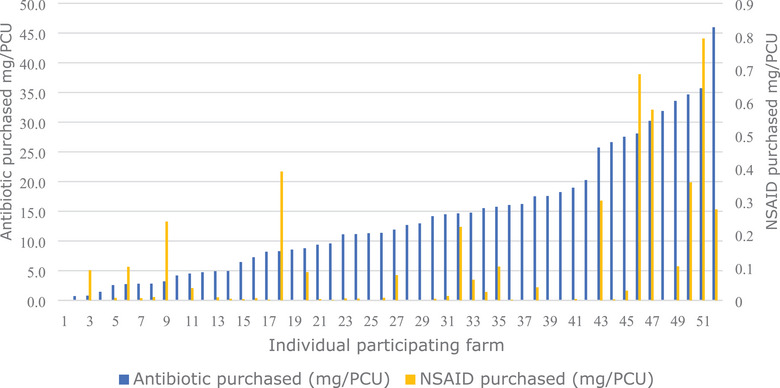
Quantity of antibiotic and non‐steroidal anti‐inflammatory drugs (NSAID) identified in the medicine purchase records supplied by 52 Northern Irish sheep farmers and standardised to account for the varied flock sizes using the mg/population‐corrected unit (mg/PCU) metric.

All antibiotics purchased were identified from the records and analysed. Following calculation of the mean antibiotic consumption for each flock, in mg/PCU using the University of Nottingham Antibiotic use calculator,^23^ this was compared with NSAID purchase calculated in mg/PCU (Figure 2) to illustrate the wide range of both antibiotic and NSAID purchase between farms, having accounted for flock size. The relationship between NSAID and CS purchases, with each of total flock size, total flock antibiotic consumption and standardised antibiotic consumption (mg/PCU) per sheep, were tested for significance using Spearman's R.^25^ No significant relationship between any pairs was observed (Table 2).

**TABLE 2:** Significance of association between flock size and antibiotic use on levels of non‐steroidal anti‐inflammatory (NSAID) use and corticosteroid (CS) use and between the level of NSAID and CS use on Northern Ireland farms supplying medicine records.

Relationship	Spearman's R, *r*_s_	*p*‐Value (two‐tailed)
Flock size versus NSAID (*n* = 52)	−0.134	0.35
Flock size versus CS (*n* = 52)	−0.058	0.68
Mean antibiotic purchase versus NSAID (*n* = 52)	0.264	0.06
Mean antibiotic purchase versus CS (*n* = 52)	0.153	0.28
Total antibiotic purchase versus NSAID (*n* = 52)	0.094	0.51
Total antibiotic purchase versus CS	0.0408	0.77
NSAID versus CS (*n* = 52)	0.338	0.014^a^
NSAID versus CS (*n* = 41)	0.077	0.63

^a^ Only the relationship between NSAID and CS use appeared significant; however, when the 11 non‐users of both medications were excluded, there was no significant relationship.

### Interviews and discussion groups

The mean farmer interview duration was 46 min (range 20–74 min) and the mean veterinarian interview duration was 55 min (range 31–84 min) (Table 3). The interviews and discussion groups covered a wide range of topics pertaining to medicine use on the sheep flock—only information related to pain perception and management, and the purchase and use of NSAID and CS is reported here. Additional quotations are provided in the Supporting Information S2.

1 Farmers demonstrated an understanding that some of their sheep suffered from painful conditions and that not only could they benefit from some form of pain relief, but that provision of an anti‐inflammatory was an essential part of the sheep's treatment.

SF65—To treat contagious digital dermatitis, everything had to be given—meloxicam as well as an antibiotic. I also used it to treat ewes with peri‐orbital dermatitis. A simple antibiotic cured the infection but to stop the irritation, they needed meloxicam as well; otherwise, they just kept rubbing and rubbing their face until one did so much damage, and she had to be euthanased.
DG02—A caesarean or something like that there I would give it to them for a couple of days—it definitely helps. I think it is necessary that you do give them something for the pain.

2 Some farmers chose to pre‐emptively purchase NSAID as part of their lambing preparations, so they would have the medicine to hand if it was needed.

SF30—Before lambing, I go to my veterinary practice with a shopping list: bottles of calcium and antibiotic injection and some sort of anti‐inflammatory and some sort of painkiller. I like having them in the cupboard. Then, if you have problems around lambing, an ewe with mastitis, for example, she is going to get pain relief.

3 This awareness or use of pain relief was not universal and some farmers indicated that some sheep received analgesia, but not all.

SF33—Sheep with contagious ovine digital dermatitis (CODD) have been proving very difficult to get cured. You get to a certain level of improvement with them. I have been using Alamycin LA (oxytetracycline 200 mg/mL, Norbrook Pharmaceuticals) for that and a wee bit of Hexasol (oxytetracycline 300 mg/mL combined with flunixin 20 mg/mL, Norbrook Pharmaceuticals) for pain, if you thought they were needing an anti‐inflammatory.

4 Farmers generally indicated that their veterinarian had initially recommended the use of NSAID.

DG02—My veterinarian's recommendations got me started using painkillers—you would notice a difference. You maybe did not have to use as much antibiotic actually. The painkiller was maybe actually doing more good than the antibiotic was what you were finding sometimes.

5 Veterinarians were positive about the benefits of providing pain relief for sheep following difficult lambings, surgery and when asked to prescribe treatments for conditions they considered to be painful. They also relayed feedback from farmers about the efficacy of the pain relief. Mastitis and lameness were the two disease conditions most frequently identified by them as benefitting from analgesia.

V11—I like everything that is assisted during lambing to get meloxicam. Everything. Also, anything with mastitis, anything that is lame. I am a big NSAID advocate. Painkillers are key.

6 One veterinarian observed that farmers were surprised at how effective meloxicam was at reducing straining in sheep who had suffered a vaginal prolapse. Additionally, veterinarians had also observed that their farming clients appreciated the benefits of the pain relief provided and that farmers now came into the practice looking for pain‐relieving products with sales increasing over time.

V20—Farmers say ‘I want to come in and have a chat to you before lambing’. They come to get their lube, gloves, antibiotics, pain relief and their colostrum. The use of NSAIDs is a massive improvement in the last 10 years, and the number of sheep that get pain relief compared to years ago—there are many more sheep getting them.

7 A wide range of factors were suggested for this increased demand from clients for analgesic products, including the influence of social media, animal welfare campaigns, greater education on the issue and veterinarians talking to their clients. Some suggested that younger farmers and female farmers would be the first ones to give pain relief and ask about what options existed to improve pain management in their flocks. However, one veterinarian noted that they were only getting through to a few farmers and that there were lots of sheep farmers that they never engaged with, as they did not seek veterinary advice. Another, while specifically mentioning cattle, discussed how he felt some farmers administer some form of medication to a non‐specifically dull animal ‘just in case’ it was to deteriorate overnight and they encouraged farmers to consider using an NSAID rather than an antibiotic. An unrelated sheep farmer who had adopted such practice was positive about it.

DG—You don't have to use as much antibiotic actually. I have found an anti‐inflammatory was as good as the antibiotic, you know. The painkiller was maybe doing more good than the antibiotic was, I was finding.

8 Some veterinarians highlighted uncertainty in the mind of their farming clients as to which products contained a pain‐relieving component and which pain‐relieving product would be best for their sheep.

V10—There are a lot, especially of sheep farmers, that think there is a painkiller in Pen Strep (procaine penicillin 200 mg/mL, and dihydrostreptomycin sulfate 250 mg/mL, Norbrook Pharmaceuticals), for example, so it is trying to get them to understand that an anti‐inflammatory, pain relief drug is a separate product to an antibiotic.

9 The lack of a product containing an NSAID authorised for use in sheep was identified by prescribers, with some showing awareness of the need to apply a statutory withdrawal.

V15—Painkillers is a difficult one because there is nothing licensed out there. We would use meloxicam.
V20—I know maybe it is not licensed in sheep, (but) we use away at the flunixin and meloxicam products. When we are prescribing it, we would put a 28‐day meat withdrawal on it.

**TABLE 3:** Participants’ demographics for the dataset addressing medicine use in the Northern Ireland sheep flock.

	Farmers	Veterinarians
Interviews	Twenty‐seven farmers, farming in five of the six counties of Northern Ireland. Farmers represented flocks of 40–730 breeding sheep. Sixty‐three percent had other livestock enterprises. Twenty‐four recorded electronically. Three recorded by contemporaneous notes.	Fifteen veterinarians from 12 veterinary practices based in Northern Ireland, who serve sheep‐farming clients across all six counties of Northern Ireland. All recorded electronically.
Discussion groups	Thirteen discussion groups with average 14 (range 8–25) participants. Farmers participating were predominantly male and over 50 years of age. Five electronically recorded. Eight recorded by contemporaneous notes.	Two discussion groups of five and seven participants, drawing veterinarians from 10 veterinary practices based in Northern Ireland, who serve sheep‐farming clients. Both electronically recorded.

Corticosteroids were only mentioned during the interviews by one farmer who indicated he had previously used them, with limited success, as part of a protocol to treat lambs with joint ill.

10 No veterinarian advocated, during interview or discussion group, a role for the use of dexamethasone in the management of painful conditions in sheep. Through the interviews and follow‐up email question veterinarians indicated that they may use it on a case‐by‐case basis where the diagnosis was unknown. While some indicated that there was a demand from their clients for access to the product, others could not identify a reason why a farmer would require CS to be supplied by the bottle.

V12—Invariably, farmers who come to the desk describing vague signs and reluctant to present the sheep for clinical examination, will leave with some sort of cortisone if they are not in lamb, you check if they are not in lamb, and a vitamin and probably an antibiotic—penicillin type or oxytetracycline based, are my favourites for those sheep.

## DISCUSSION

While Northern Ireland sheep farmers participating in this study expressed positivity about the benefits of pain relief medicines and the use of some form of anti‐inflammatory was widespread, it was not universal. This provides an opportunity for the industry to consider the barriers to use of NSAIDs and to promote their use to unengaged farmers.

To manage pain effectively, first the pain must be recognised and then an efficacious medicine must be administered.^4^ The farmers already using NSAIDs and veterinarians in this study were clear about which conditions they considered to be painful. The range of conditions that both groups identified (obstetrical, surgical, mastitis and lameness cases) reflected previous reports on ovine conditions benefitting from analgesia.^11^, ^26^ The farmers recognised the benefits that analgesia provided in aiding recovery and fed this information back to their veterinarians. Observing this response to treatment influenced farmers’ behaviour, resulting in them choosing to maintain a supply of NSAIDs on farms so there was some available immediately if needed, especially during the lambing period. This change hopefully improved animal welfare and will encourage more farmers to follow suit, given the significance of peer influence across a range of farming situations.^27^

Farmers indicated that it was their veterinarian who had initially suggested using an NSAID as part of treatment protocols, reinforcing the previously stated view that veterinarians are trusted advisors to farmers.^21^ One veterinarian had identified a need within farmers to do something when faced with a cow who was dull (and no specific diagnosis could be reached). His approach was to encourage farmers to use NSAIDs rather than antibiotics as the first‐line treatment. This first‐line use of NSAIDs was reflected in the comments from a farmer, who, when faced with a dull sheep, realised that pain rather than infection may be the cause of the dullness and inappetence and reported a positive outcome using NSAIDs. This is an example of how past behaviour may influence attitudes moving forward,^28^ with sheep farmers electing to use NSAIDs promptly in future cases and talking openly to their peers about the benefits they perceive during discussion group sessions. Encouraging more farmers to assess their sheep and consider the potential benefits of analgesia rather than immediately reaching for antibiotics could improve the stewardship of antibiotics through the creation of a new behavioural pattern. In time, the use of pain relief as a first‐line treatment could come to define what constitutes a ‘good farmer’.^29^

Over a quarter (27%) of participating farmers were not using NSAIDs. Insufficient data were collected to definitively say whether this was due to a lack of clinical indication for analgesics, although the quantity of antibiotic purchased by some would suggest clinical infectious disease was present. Few, if any, sheep suffering from an infectious condition will not benefit from NSAID provision. Overall, while no significant relationship was identified between antibiotic and NSAID purchases, the results suggested a potential trend that may have been statistically significant with a larger dataset. Further study is needed to characterise the conditions being treated with antibiotics and the barriers to those sheep concurrently receiving analgesia.

Areas where further advice on pain recognition and treatment is potentially warranted and highlighted in these results include lameness, CODD specifically and neonatal lambs. When treating CODD, a farmer stated that ‘if you thought they were needing an anti‐inflammatory’ (SF33), they used a combination antibiotic and anti‐inflammatory product, while most of their lame sheep were treated with an antibiotic only. This, combined with the low level of use of NSAIDs on some farms purchasing significant quantities of antibiotics, suggests that there is a pain hierarchy, where some lame animals are deemed worthy of analgesia and others are not. This finding reinforces the need for farmer and veterinarian education on pain recognition and the development and dissemination of pain‐scoring tools validated for general application in an on‐farm environment.^30^ Given that only minimal reference was made by farmers and veterinarians to the provision of analgesia for routine husbandry procedures such as tail‐docking and castration, this is another area that may benefit from further engagement, given the calls for improvements in the welfare of lambs undergoing such procedures.^4^, ^30^, ^31^

Three NSAIDs were supplied by veterinary practices. None are authorised in the UK and thus defined doses were not available, complicating comparison between the relative proportions of each product supplied and in determining the number of sheep likely to have been treated based on the volumes sold onto each farm. NSAIDs are recognised as providing some analgesia in sheep.^4^, ^18^, ^26^ However, authoritative efficacy studies of NSAID use in clinical cases in the field are lacking.^26^ Veterinarians stated concerns about the lack of an authorised product for pain management in sheep and may lack the confidence to determine the optimal product and dose, to use for providing analgesia in sheep. The lack of an authorised product will inhibit promotion of NSAIDs through avenues such as professional publications. In the human medical field, advertising prescription‐only medicines directly to the public has been shown to significantly increase patients’ confidence in talking to doctors about their concerns and specific solutions.^32^ If farmers fail to see promotion of named analgesics, they may wrongly assume that, as there is no promotion of pain‐relieving medication, pain is not a welfare concern in sheep.

Meloxicam, the most commonly supplied NSAID, was described by veterinarians as their standard treatment or a preference based on experience. This product is authorised in some countries outside the UK, for use in sheep at 1 mg/kg.^33^ This is twice the authorised dose that UK veterinarians are familiar prescribing for cattle. They were not asked specifically what dose of each product they prescribed in this study. This avoided participants feeling that they were being tested on clinical knowledge, which may have reduced their trust and thus openness in participation.^34^ There remains a knowledge gap surrounding the exact dosing strategy recommended by veterinarians and how they determine the dose, treatment frequency, duration and withdrawal periods of these products.

Pressure should continue to be put on the pharmaceutical industry, or state action taken, to provide safety and efficacy data on efficacious analgesia products for sheep. Veterinarians could then use this information to formulate prescribing protocols for all sheep, specifically neonatal lambs, to facilitate progress in analgesia provision. Additionally, an authorised product would have a specific sheep meat withdrawal period, removing the need to apply the statutory sheep meat withdrawal period, 28 days in this instance, required under the cascade.^15^

Comparative studies have failed to demonstrate any analgesia provided by dexamethasone.^35^ It has been reported that CS can have a positive effect on ameliorating clinical signs, such as inappetence and pyrexia, as well as improving the general wellbeing of a sick animal,^19^, ^36^ without improving clinical outcome except in limited, specific conditions.^19^ Responses from veterinarians and farmers in this study indicated that some continue to use CS for such purposes despite a lack of evidence of efficacy in sheep. However, where pain is the cause of dullness or inappetence, a greater impact on animal welfare may be gained through encouraging these farmers to administer an appropriate dose of an NSAID instead of the CS. Further work is needed to understand why farmers are requesting veterinary practices to prescribe CS products, which were authorised in cattle and only to be used in sheep under the cascade. It may be that farmers have followed some veterinarians’ use of these products where a diagnosis is unknown or other rational treatments have failed; alternatively, veterinarians still supply them, failing to appreciate the superior analgesic benefits NSAIDs can provide. As CS are known to induce parturition, they are contraindicated where the pregnancy is to be maintained. This may mean sheep miss out on analgesia for conditions such as lameness, where a farmer relies on CS, but knows not to administer them to pregnant ewes. In New Zealand, where meloxicam is authorised for use in sheep, the product carries no contraindications for use during pregnancy^33^ and may offer safe and efficacious analgesia for sheep throughout pregnancy.

Quality assurance schemes often require farmers to review antibiotic use.^37^ The disparity in NSAID use between farms suggests that a review of analgesia provision is also important to improve animal welfare. The lack of authorised dosages, differing potencies and concentrations of active ingredients in NSAIDs makes the analysis and benchmarking of these products complicated. If suitable metrics could be developed and validated, then an objective review of the farmers’ provision of pain relief could be instigated.

Veterinarians should ensure that when they attend to a sheep suffering from a painful condition and provide analgesia, they could discuss which product they have dispensed and why with the farmer.^30^ Veterinarians participating in this study highlighted that farmers are not always aware of the purpose of medications administered to their animals. This will allow farmers to learn about and appreciate the benefits of pain relief in sheep, hopefully going on to adopt analgesia into their normal routine practices. However, one veterinarian here highlighted that such opportunities to interact with many sheep farming clients can be rare because these clients tend to be self‐sufficient, emulating previous observations about sheep farmers.^38^

Veterinarians and farmers highlighted that veterinary consultation over the best method of treating animals was central to farmers adopting NSAIDs for pain relief in their flocks. Clear efficacy and safety data should be developed to enable wider promotion of analgesia for sheep. To further promote animal welfare, new approaches need to be considered to encourage more engagement between farmers and their veterinarians. Until suitable metrics have been developed for benchmarking NSAID use, a subjective approach can be adopted. Veterinarians should be encouraged to challenge farmers each time antibiotics are prescribed, both on whether the antibiotic is necessary for the condition and whether analgesia is also required.

## AUTHOR CONTRIBUTIONS

All authors contributed to the design of the wider project from which these data are drawn. Paul E. Crawford conducted all data collection and analysis. All authors contributed to the writing and critical review of the manuscript.

## CONFLICTS OF INTEREST STATEMENT

The authors declare they have no conflicts of interest.

## ETHICS STATEMENT

The authors confirm that the ethics policies of the journal, as noted on the journal's author guidelines page, have been adhered to. Ethical approval was obtained from the Research Ethics Committee of Harper Adams University (approval number: 0010‐202101‐PGMPHD).

## Supporting information

Supporting Information

## Data Availability

Due to the confidentiality agreements entered between researcher and participants, the raw data cannot be made available.
