# Supplementary material for: Improving analgesia provision for sheep: An analysis of farm medicine records and attitudes towards pain relief on sheep farms in Northern Ireland
Source: Vet Rec Open. 2023 Oct 23;10(2):e75. doi: 10.1002/vro2.75 (PMC10591906; doi:10.1002/vro2.75)
Supplement: Supplementary file 1 — Supporting Information [file VRO2-10-e75-s001.pdf]

Supporting Information

S1 - Questionnaires

Phase 1a Farmers The interview

Recording medicine use	• Types of information recorded;<br>• Methods of recording<br>• What proved difficult / impossible to record;<br>• Veterinarian, Pharmacist, Suitably Qualified Person (Prescription Only Medicine - Veterinary, Pharmacist, Suitably qualified person) medicines' recording<br>• Searchable<br>• Time spent recording;<br>• Business benefits of recording;<br>• Personal satisfaction benefits;
National recording and benchmarking	• Metrics<br>• Benchmarking
Number of medicine sources	• Relationship with supplier<br>• Prescribed or sold<br>• Reasons for multiple sources<br>• Do each know of the other's involvement in managing farm stock health<br>• Do you declare all in the medicine record book?
Demographic, geographic and educational information, continued development / training.	• Education, agricultural, other.<br>• Recent antimicrobial training – MilkSure, College of Agriculture, Food and Rural Enterprise, local veterinary surgeons,<br>• Farm Quality Assurance Scheme, Ulster Farmers' Union, National Sheep Association
Medicine use	• What when why how<br>• Health plan's influence
National initiatives; future direction	• Existing<br>• Feared<br>• Hoped for

Phase 1b   Prescribers   The interview

Protocol for sales	• Agreed plans on file<br>• Generalised dispensing guide for lay staff<br>• Questioning reason for medicine request<br>• Follow-up diagnostic / preventative<br>• Requests presented over the counter to a person other than a qualified prescriber<br>• Farm stock level knowledge
Different approaches for specific antibacterials & indications / uses,	• Critical antibiotics<br>• Oral products for neonatal lambs;<br>• Antibiotic injection for abortion,<br>• Soluble power for footbath
Understanding & interpretation of Royal College of Veterinary Surgeons / Veterinary Medicines Directorate etc. guidelines	• Under care<br>• Competence for safe use<br>• Questions that should be asked
Role of / place for national treatment guidelines	• Sustainable Control Of Parasites in Sheep<br>• Lameness 5 Point Plan<br>• British Small Animal Veterinary Association equivalent
Personal and practice/business level involvement in national training schemes	• MilkSure,<br>• Farm Vet Champions<br>• Your knowledge?
Beliefs about farmers knowledge when requesting drugs	
Services offered, uptake	On farm
Belief and frustrations about (in)ability to influence farmer behaviour	Carrots and sticks
National initiatives; future direction	• Existing<br>• Feared<br>• Hoped for
Spectam (spectinomycin 50mg/ml oral solution, Ceva Animal Health) – did you see an effect?	

One follow-up question was sent by email to participating veterinary surgeons with a brief introduction to explain the request and a reassurance that the confidentiality of the participants would be respected.

When interviewing and participating in discussion groups, little mention was made by vets of corticosteroid use in sheep but when I reviewed the farmer supplied medicine records they appear in half of these record.

Why are these drugs prescribed, or are they requested by farmers?

As ever, anything you say will be confidential and anonymised before reporting in my thesis and other outputs. Also, happy to have a chat on the 'phone / zoom if you prefer.

Paul.

Discussion groups (farmer)

Introduction<br>FLOCK AND FARM	Around the table briefly introducing one another and the farms and flocks we manage	• Location<br>• Area farmed<br>• Type of ground<br>• Size and structure of flock<br>• Other enterprises	10 minutes
<u>Discussion one</u><br>Problems and priorities	Around the table list flock health related problem<br>Then prioritise these as a group	No guidance, hints given at this stage	10 minutes
	Consider has everything been addressed including the non-clinical threats	Prompt if necessary.<br>- Access to vet services, economics, labour, 'iceberg' diseases, unpick vague concepts such as bio-security if raised	10 minutes
<u>Discussion two</u><br>Possibilities and solutions	A - What have you tried and what have you not tried, why?	Work down the problem list<br>Asking about practicality, sustainability etc of the solution elements	20 minutes
	B - What are there no solutions for? What is needed 'blue sky'.	In particular focusing in on any areas that it is felt there is no practical solution	10 minutes
<u>Discussion three</u><br>Others say this is a problem	• Lack of data<br>• Lack of control on medicine availability / multiple sources of medication<br>• Unwillingness to ask / pay for advice proactively	Response & potential mitigation / solution(s)<br><br>Potentially these may have been addressed already	20 minutes
Concluding comments	Ask for any further important suggestions		10 minutes

Vets

Thank for help in interviews, recruiting farmers and supplying records.

<u>Highlights of research to date</u>

- Medicine use – patterns, low versus high, prescribing patterns, treat rather than prevent, lameness ongoing issues, pockets of progress, metrics.

- Flock health plans or flock health planning.

<u>Questions / discussion points</u>

- Resources – vets, impact of part-time farming, to deliver flock health planning and prescribing, testing and prescribing of anthelmintics.

- Resources – Information technology systems to manage prescribing.

- One-farm-one-vet? or Central prescription register or what?

- Unlicensed drugs – recurrent sales.

<u>Successes</u> transition to vaccine over antibiotic for abortion, pain-relief.

Farming representatives

Thank you for agreeing to host and facilitate a Zoom for my PhD with some of your colleagues.

Agenda is short
1. I present findings.
2. We discuss next steps for the industry.

In slightly more words and to give a little context and background:

I plan to highlight research findings to date around:

i. Medicine use
ii. Lack of oversight in medicine (antibiotic) supply
iii. Flock health planning
iv. Farm Quality Assurance Scheme – opinions of and behaviour relating to inspections and record keeping
v. Medicine recording on-farm use
vi. Impact of (non-disclosure of) sourcing medicines from multi-vet practice
vii. Perceived lack of incentives in sector to progress
viii. Ongoing lameness problem, low awareness of control plans or even causes
ix. Gaps in research base

From a positive standpoint:

x. Reduction in antibiotic for abortion already achieved and
xi. The world carried on when Spectam (spectinomycin 50 mg/ml oral solution, Ceva Animal Health) disappeared - showing the industry can respond positively to animal health messages. (This product, the final authorised oral antibiotic for neonatal lambs in NI, was suddenly withdrawn from sale shortly before the 2022 lambing season – the lambing season immediately prior to the discussion groups taking place.)

Any other bits you think I should be asking questions about but have not done so to date.

Seek feedback on what industry might think are appropriate ways to progress any of these areas (these following options are not my recommendations at this point but are options that have been raised by others and or are in use elsewhere)

i. The suggestion that there should be a regulation meaning each farmer has to register at only one veterinary practice – '1 farmer - 1 vet'.
ii. Improved medicine recording [currently a statutory requirement for all, not just a Farm Quality Assurance Scheme standard, but not enforced to any degree]
iii. Improving oversight of prescription of medication (antibiotic)
iv. Prescription register [which any certifying vet would have access to a record of all medicine purchased]
v. Test results required to prescribe anthelmintics
vi. Future farm support tied to participation in schemes; Southern ewe premium, English Pathway, Scottish… not got a name yet but one suggestion is payment linked to data-recording, discussion, and knowledge exchange events.
vii. Where do Business Development Groups (or their successor) sit in all this?
viii. Incentivising and resourcing farmers – doing this without ignoring the ones who have already taken steps to improve
ix. Diagnostic testing facilities
x. Improved screening of livestock entering markets

Any other thing you guys want me to consider?

Other industry reps

As above with specific question below for those involved in quality assurance.

Quality assurance – use of shower systems; what goes in the medicine book is not all that goes in the sheep; lack of incentive means [small] issues with inspections / inspectors means farmers throw the head up; lack of progress on lameness.

S2 Additional quotes from interviews and discussion groups

1. Farmers holding a stock of medicines in preparation for lambing.

SF52 Before lambing time, you stock up on your stuff that you might need. You would keep a bit of meloxicam sort of thing, just for pain relief, especially if it was a hard lambing or Pen Strep [penicillin streptomycin combination antibiotic, Norbrook Pharmaceuticals] or something like that there for infections. Not just counting on Pen Strep to work all the time.

2. Farmers' use of painkillers.

Interviewer: Painkillers, what about them?
SF72 What do you mean painkillers? For myself?
SF69 Whenever a ewe gets mastitis the one thing you don't give her is the pain relief and the anti-inflammatory. We give her the antibiotics and not the pain relief.

3. Vets influenced farmers' adoption of NSAID.

DG01 I went and asked the vet for something for a sheep with a particular condition and he gave me some antibiotic and the anti-inflammatory as well. I noticed they got better quicker.

4. Farmers shared their positive experiences of NSAID to other farmers.

One farmer (SF61) indicated that they had encountered the benefits of meloxicam when reading an article in a farming magazine where another farmer was advocating its use, alongside an antibiotic, to improve cure rates for mastitis in sheep.

5. Vets appreciated that NSAID provide clinical benefits.

V12 Our standard practice is that anything we lamb or has a caesarean-section, all get pain relief. The ewes that get the pain relief, when the farmer is in again, they tell us, 'Ah, she never looked back, she wasn't sore, she took the lamb, she was happy to let them suck.' And, again with feet, you know, farmers they do see a benefit or a merit in pain relief. That message filters down.

V13 CODD is excruciatingly painful and I think it is a bigger welfare issue than footrot because sheep with it have pretty horrific lameness.

6. Vets also identified NSAID could be used preferentially to antibiotics where there was not a clear-cut indication that an antibiotic was needed, but a farmer wanted to 'try something' rather than present the patient for examination.

V13 I think it is a feeling that they have to do something. And I think it is always the cow late at night that doesn't look quite right. They have looked at her all day and they are going to leave her now for maybe 10 hours. 'I'd better give her a jag [injection] here to keep her right overnight.' By all means, give her a jag of non-steroidals, but you don't always need to give her a jag of antibiotics.

7. Vets recognise farmers appreciated the benefits of providing analgesia for their sheep; this was a change in attitude experienced vets had observed developing over many years.

V13 I think that over my career there are definitely a lot more non-steroidals used in sheep now than there would have been 30 years ago. Farmers will get a bottle of non-steroidals pre-lambing and use meloxicam readily for sheep that have had a hard lambing. That is something positive.

V19 I would say people are more inclined to ask for painkiller so there is progress in that direction. We would always routinely have given them antibiotic and a painkiller for something like mastitis, but when they come in wanting to make their own decision, you would find that now they are more inclined now to ask for the anti-inflammatory. A lot of the farms, the bigger farms, would keep a bottle on site. Matter of routine, so I suppose that is something you wouldn't have seen 10 years ago.

V20 I'd say social media, animal welfare, all the far right, education, a lot of things, vets talking to them, there is a whole lot of factors that have influenced the uptake of pain relief. Younger farmers I would imagine

too, female farmers. There are a lot of females involved, they would be the first ones to give pain relief and talk about a lot of them will ask you what's the best way to ring a lamb's tail or lose his tail with least pain. Females I find are very good at that.

8. Vets recognised that not all farmers knew which medicines provided analgesia:

V06 A lot of them in my opinion, don't see the need for pain relief or they think the only pain relief out there is dexamethasone.

V15 We would have a few farmers who will come in and ask for steroids, and when you say 'What do you want a steroid for?' they say that it is good for pain and makes the sheep eat as well. However, I find the meloxicam seems quite good, lasting longer than steroid after a single injection, they get might 36 - 48 hours cover, so even after difficult lambings and that we would probably recommend using meloxicam.

9. Vets were unsure why sheep farmers would purchase dexamethasone by the bottle, as they saw only occasional indications for it.

V02 I wouldn't have thought it would be something that farmers would require a bottle of or would particularly ask for. Things I would use it for are: when inducing parturition in a ewe, especially if she has had pregnancy toxaemia. Some severe cases of mastitis or pneumonia, joint ill and allergic reactions. In lambs I would use it for those noisy after birth or have possibly aspirated and those with fractured ribs at birth. More generally, where there is an inflammatory issue and I also would like to improve appetite.

V15 Farmers request and use corticosteroids for pain relief and also view them as an appetite stimulant.

Table S1 Frequency distribution of quantities, by volume per breeding ewe, of each non-steroidal anti-inflammatory drug (NSAID) and corticosteroid (CS) purchase, by farmers, as identified in the twelve-month sample of medicine records supplied relating to 52 Northern Irish sheep farms.

		**None**	**Less than 0.025 ml**	**0.025-0.05 ml**	**0.05-0.1 ml**	**0.1-0.25 ml**	**0.25-0.5 ml**	**0.5-1 ml**	**1-2.5 ml**	**2.5-5 ml**
NSAID	Meloxicam	17 (33%)	9 (17%)	7 (12%)	3 (6%)	3 (6%)	3 (6%)	4 (8%)	4 (8%)	2 (4%)
	Ketoprofen	50 (96%)	0 (0%)	0 (0%)	0 (0%)	1 (2%)	0 (0%)	1 (2%)	0 (0%)	0 (0%)
	Flunixin	46 (88%)	2 (4%)	1 (2%)	0 (0%)	2 (4%)	0 (0%)	1 (2%)	0 (0%)	0 (0%)
CS	Dexamethasone	26 (50%)	3 (6%)	4 (8%)	6 (12%)	6 (12%)	4 (8%)	1 (2%)	2 (4%)	0 (0%)

No NSAID or CS purchases were identified in eleven records; twenty-four records indicated purchase of both NSAID and CS. Four showed purchased of more than one NSAID.
